# Supplementary material for: Obtaining 2,3-Dihydrobenzofuran and 3-Epilupeol from Ageratina pichinchensis (Kunth) R.King & Ho.Rob. Cell Cultures Grown in Shake Flasks under Photoperiod and Darkness, and Its Scale-Up to an Airlift Bioreactor for Enhanced Production
Source: Molecules. 2023 Jan 6;28(2):578. doi: 10.3390/molecules28020578 (PMC9865622; doi:10.3390/molecules28020578)
Supplement: Supplementary file 1 [file molecules-28-00578-s001.zip › molecules-2065493-supplementary.docx]

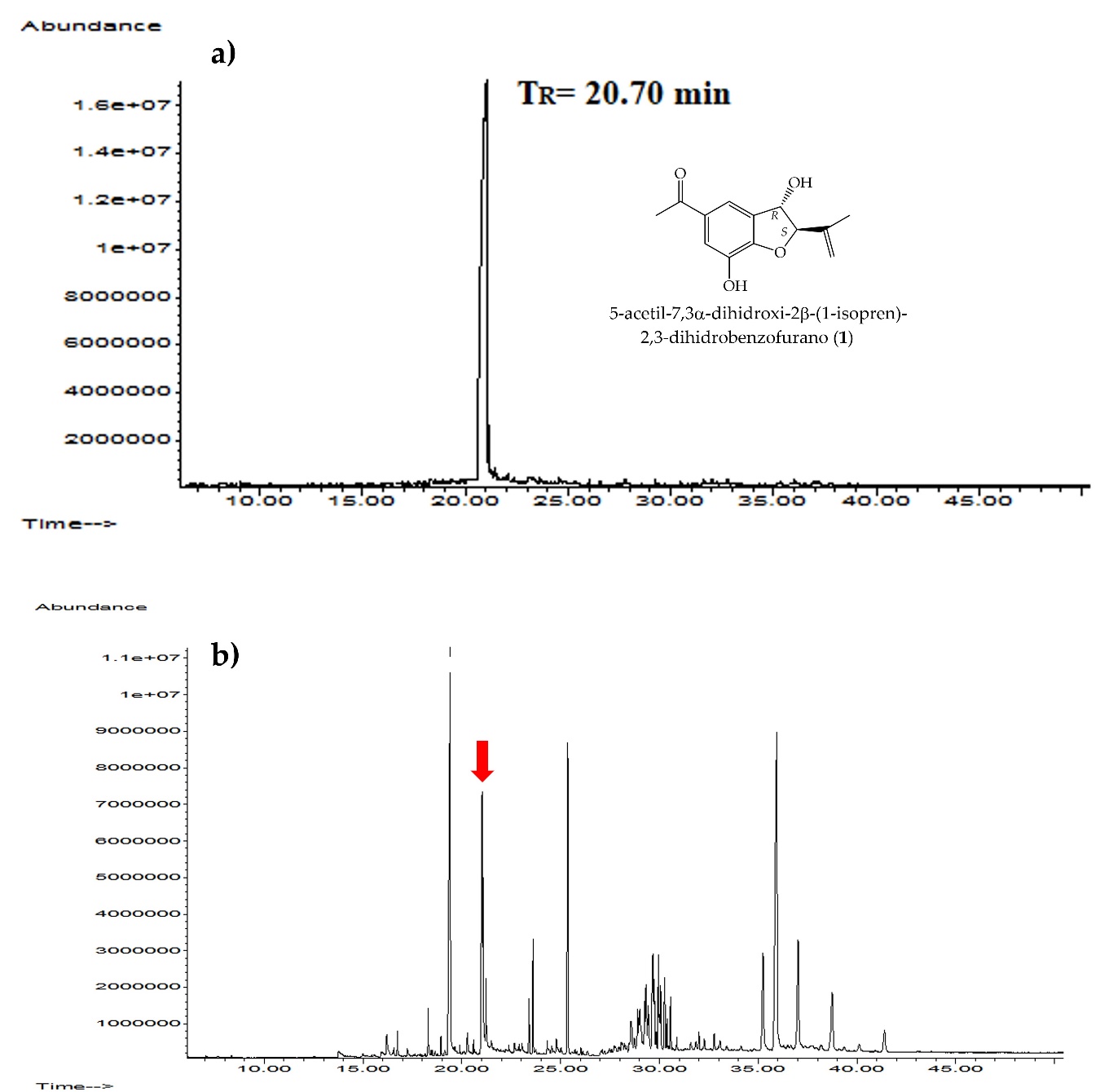


**Figure S1**. GC-MS chromatograms for standard compound and EtOAc extract. **a**) 2,3-dihydrobenzofuran profile used a standard; **b**) EtOAc extract profile from *A. pichinchensis* cell culture suspension cultivated in an airlift bioreactor at 7 days of culture showing the peak of 2,3-dihydrobenzofuran.


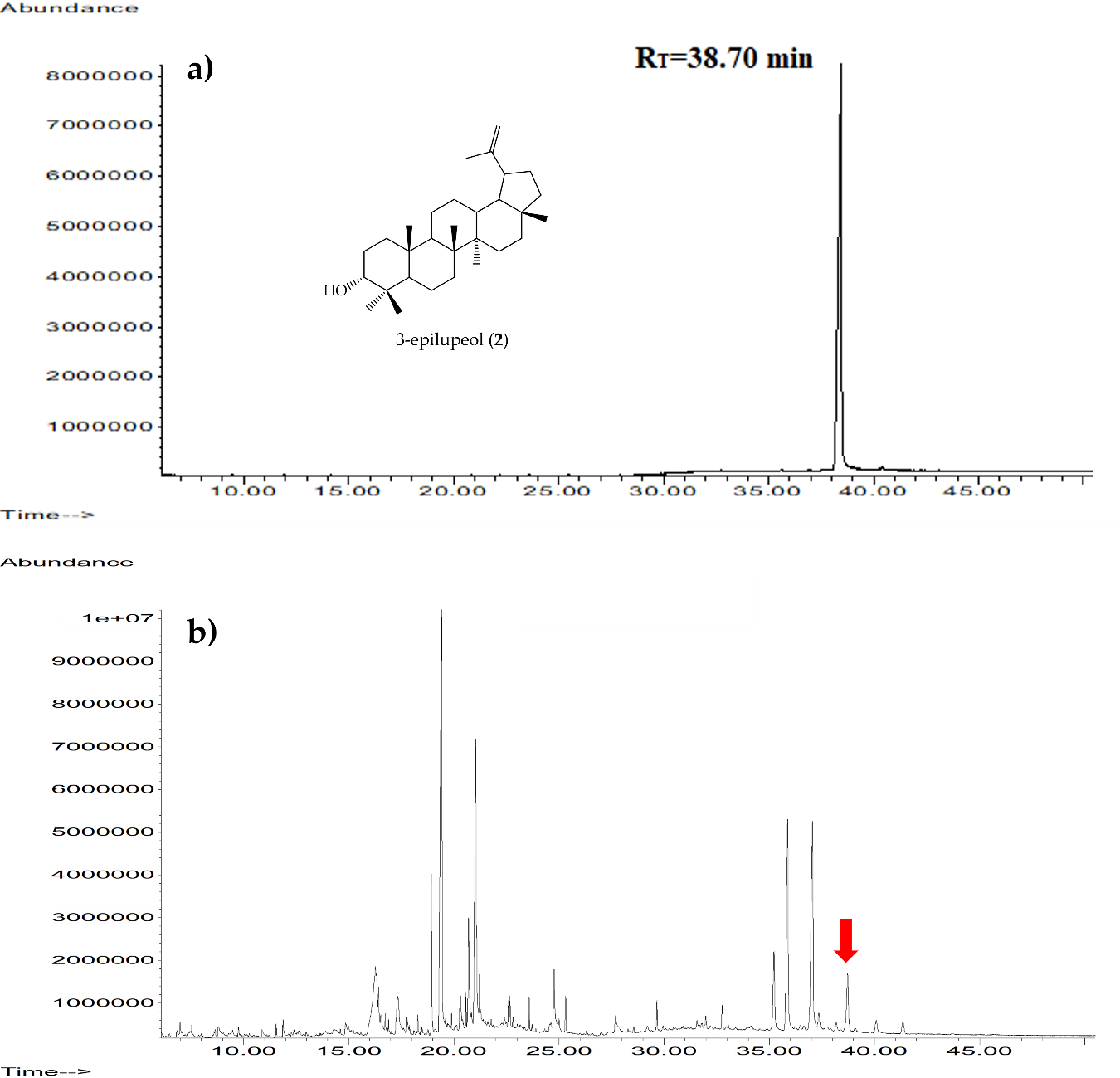


**Figure S2.** GC-MS chromatograms for standard compound and EtOAc extract. **a**) 3-epilupeol profile used a standard; **b**) EtOAc extract profile from *A. pichinchensis* cell culture suspension cultivated in an airlift bioreactor at 14 days of culture showing the peak of 3-epilupeol.
